# The Effect of 75 Grams of Glucose during OGTT on Plasma Markers of Lipid and Lipoprotein Peroxidation, Oxidized LDL and Thiobarbituric Acid Reactive Substances, in People with Increased Body Mass

**DOI:** 10.3390/metabo13040483

**Published:** 2023-03-27

**Authors:** Lena Bielawska, Ewa Wysocka, Aleksandra Baszczuk, Anna Dżumak, Aleksandra Ludziejewska, Maciej Cymerys, Alicja Płóciniczak

**Affiliations:** 1 Department of Laboratory Diagnostics, Poznan University of Medical Sciences, 84 Szamarzewskiego Str., 60-569 Poznań, Poland; 2 Department of Internal Medicine, Metabolic Disorders and Arterial Hypertension, Poznan University of Medical Sciences, 16/18 Grunwaldzka Str., 60-780 Poznań, Poland

**Keywords:** lipoproteins, oxidative stress, insulin resistance, obesity

## Abstract

Obesity, currently defined as a disease, is associated with a number of metabolic disorders, and oxidative stress is discussed as the link between them. The aim of this study was to analyze the plasma markers reflecting oxidative modification of lipids and lipoproteins, oxidized LDL (oxLDL) and thiobarbituric acid reactive substances (TBARS), under the influence of the 75 g of oral glucose during oral glucose tolerance test (OGTT), in patients with increased body mass. One hundred twenty individuals of both genders (46 women and 74 men) aged 26 to 75 years with increased body mass (BMI > 25 kg/m^2^) were recruited for the study. OGTT was performed in each of the qualified persons, and glycemia, insulinemia, and concentrations of oxLDL and TBARS were measured fasting and at 120 min of OGTT. The homeostasis model assessment of insulin resistance (HOMA-IR) was used to assess the degree of insulin resistance (IR). In order to assess the changes of the investigated parameters under the influence of 75 g glucose, the index R_OGTT_ = [120’]/[0’] was calculated to obtain oxLDL-R_OGTT_ and TBARS-R_OGTT_. The statistical analysis was performed in the entire study population and subsequent groups from H1 to H4, defined by HOMA-IR quartiles. In the entire study population and the subgroups, oxidative stress markers changed during OGTT. From H1 to H4 group, increasing oxLDL and TBARS were observed both in the fasting state and at 120 min of OGTT, and the oxLDL-R_OGTT_ index decreased from the H2 to the H4 group. The intensification of IR in people with increased body mass may predispose them to enhanced oxidative modification of lipoproteins. Individual reduction in the concentration of oxLDL during OGTT, in reference to fasting value (decreased oxLDL-R_OGTT_), suggests increased uptake of modified lipoproteins by scavenger receptor-presenting cells or increased migration to the vascular wall.

## 1. Introduction and Aims

Obesity, currently defined as a disease [1], is associated with a number of metabolic disorders, such as insulin resistance (IR), type 2 diabetes, atherosclerosis, dyslipidemia, and hypertension, leading directly to increased cardiovascular risk. It was estimated that the prevalence of overweight and obesity globally in 2016 was 1.9 billion adults, which is almost 40% of the population [1]. The criterion for assessing obesity is a simple indicator of BMI—body mass index. However, the BMI used in epidemiological studies in adults does not account for the high inter-individual variability in body fat content depending on age, sex, or ethnicity [2].

Oxidative stress is recognized as the link between metabolic disorders. In the course of oxidative stress, excess reactive oxygen species (ROS) interact in an uncontrolled way with proteins, nucleic acids, and fatty acids, leading to the oxidative modification of these molecules, which may result in a change in their structure and function [3]. Lipid peroxidation is thought to be the main mechanism of cell and tissue damage caused by ROS [4]. The consequence of this process is the modification of their physical properties, followed by the disruption of the integrity of cell membranes. This is due to a decrease in the hydrophobicity of the internal part of membranes and a change in the bilayer lipid structure [5]. The effect of ROS on the plasma molecules is the formation of, among others, oxidatively modified low-density lipoproteins (LDL), the presence of which is one of the most important elements of the pathomechanism of atherosclerosis [6]. Alternative plasma lipoprotein modification mechanisms include desialylation, glycation, glycosylation, carbamylation, or glycoxidation. Small dense LDLs are particularly vulnerable to these modifications due to the lower affinity for the LDL receptor and prolonged time of circulation in blood. However, mentioned modifications are not exclusive to LDL [7]. Researchers are also interested in, for example, modified HDL particles [8,9].

In order to maintain the oxidative balance of the body, the antioxidant defense system is necessary. The first line of defense is enzymes (i.e., superoxide dismutase, glutathione peroxidase, catalase), which limit the formation of a highly reactive hydroxyl radical. Small molecule antioxidants, such as vitamin E, A, or C, as well as glutathione, coenzyme Q, and others, are responsible for its neutralization. Antioxidant defense is also supported by repair enzymes and metal-binding proteins [4,10].

Glucose metabolism disorders also play an important role. High blood glucose concentration favors the accumulation of lipoproteins modified by glycoxidation. Insulin resistance is an essential factor in the development of dysglycemia and, consequently, type 2 diabetes. Among the available methods of assessing insulin resistance, the homeostasis model assessment of insulin resistance (HOMA-IR) is the most frequently used, as it requires the determination of fasting glucose and insulin concentrations only. Its usefulness in clinical practice is limited due to the lack of precisely defined cut-off values for diagnosing insulin resistance. In the literature, we can find studies in which attempts are made to determine the HOMA-IR cut-off value [11,12].

Direct measurement of ROS, especially the hydroxyl radical, is difficult due to their instability. However, it is possible to determine various products of lipid, protein, and nucleic acid peroxidation. The effect of ROS on lipid particles can be measured by the colorimetric method and expressed as the concentration of thiobarbituric acid reactive substances (TBARS) [13]. The concentration of oxidized LDL (oxLDL) in serum or plasma can be determined by enzyme immunoassay using antibodies against oxidatively modified LDL component—apoprotein B [14]. It can be assumed that the lipid and protein parts of LDL undergo oxidative modification to a similar extent.

The aim of this study was to analyze the plasma markers reflecting oxidative modification of lipids and lipoproteins and potential factors modifying this process in patients with increased body mass, in particular, the assessment of the dynamics of changes in these parameters under the influence of the 75 g of oral glucose, the correlations between them and the impact of cardiovascular risk factors on examined parameters. The selected markers of oxidative stress in the blood were: oxidized LDL and thiobarbituric acid reactive substances.

## 2. Material and Methods

### 2.1. Study Population

The study was conducted on Caucasian people aged 26–75 with increased body mass (BMI > 25 kg/m^2^). Females and males referred to outpatient clinics of Poznan University of Medical Sciences because of their overweight/obesity were qualified for the study.

The excluding criteria were: acute and chronic diseases such as myocardial infarction, stroke, respiratory system diseases (i.e., chronic respiratory failure, pneumonia or bronchitis), acute and chronic diseases of kidney and liver, autoimmune diseases, endocrine gland hyperfunction and insufficiency, neoplastic disease, anemia and disorders of hemostasis.

**Figure 1:**
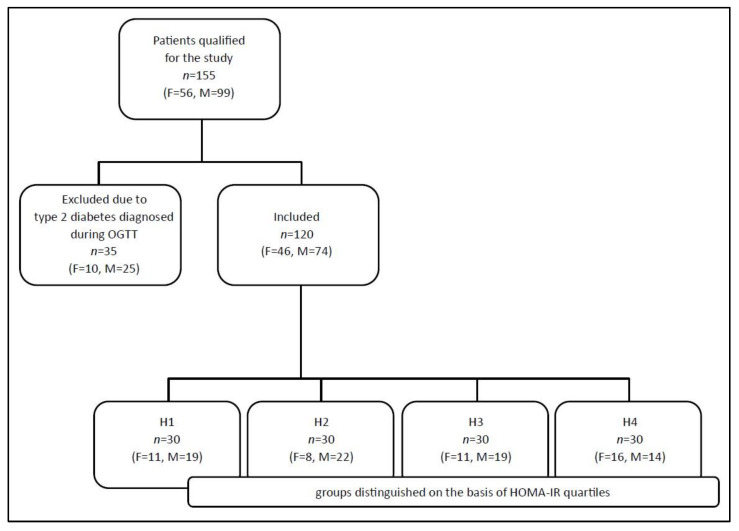
The flowchart of patient recruitment for the study (F—female, M—male).

The including criteria were: no previous diagnosis of diabetes and current fasting glucose <7.0 mmol/L, no contraindications to performing the oral glucose tolerance test (OGTT), C-reactive protein (CRP) concentration in blood <10 mg/L, and no pathological results of routine laboratory tests (i.e., basic hematology tests, liver, and kidney function tests). One hundred fifty-five subjects were initially qualified for the study on the basis of physical examination and medical documentation. Thirty-five of them were excluded because they were newly diagnosed with type 2 diabetes based on the results of the OGTT performed in the study. Finally, 120 adults (46 women and 74 men), who used no medication, no supplements, no special diet, and no alcohol, were included in the study. The flowchart of patient recruitment for the study is presented in Figure 1.

### 2.2. Measurements and Analysis

In order to assess the effect of the increase in glycemia on the level of markers of oxidative modification of plasma lipids and lipoproteins, the OGTT was used, in which 75 g of glucose was considered a stimulus. Oxidative stress parameters, oxLDL and TBARS, were determined in the subjects during OGTT: fasting (0′) and 120 min (120′) after administration of 75 g glucose.

After a medical history and physical examination, including the measurement of waist circumference (WC), systolic (SBP), and diastolic (DBP) blood pressure, OGTT was performed in each of the qualified persons (according to current recommendations of the International Diabetes Federation (IDF) and the American Diabetes Association (ADA). The test material, serum and heparin plasma, was obtained from patients’ blood collected during the OGTT. Blood was collected in accordance with current standards, both in terms of patient preparation and sampling. Fasting and 120 min after administration of 75 g glucose in the OGTT, whole blood was collected into a clotting activator tube (4.9 mL) for serum and into a heparin tube (4.9 mL) for heparin plasma (S-Monovette^®^, Sarstedt, Germany).

The concentrations of glucose, total cholesterol (T-C), HDL cholesterol (HDL-C), LDL cholesterol (LDL-C), and triglycerides (TG) were measured in fresh blood samples on the automatic biochemistry analyzer Dimension EXL (Siemens Healthcare Diagnostics Inc., Tarrytown, NY, USA) with enzymatic methods. Non-HDL (nHDL-C) cholesterol was calculated. The remaining assays were made on samples stored at −80 °C. The concentrations of plasma insulin (Ins) and oxLDL were measured fasting and at 120 min of OGTT by enzyme-linked immunosorbent assay, using DRG (DRG International, Inc., Springfield, NJ, USA) and Mercodia (Mercodia AB, Uppsala, Sweden) kits, respectively, and microplate reader TECAN (Tecan Group Ltd., Männedorf, Switzerland). The intra- and interassay coefficient of variation (CV) was 2.2% and 4.5% for Ins and 6.4% and 7.9% for oxLDL, respectively. The concentrations of TBARS during the OGTT were determined by the Ohkawa method using Sigma reagents (Germany) and spectrophotometer Specord M40 (Germany). The intra- and interassay CV for TBARS were 2.0% and 3.6%, respectively.

In order to assess the changes of the determined parameters under the influence of 75 g glucose, the index R_OGTT_ = [120′]/[0′] was calculated to obtain oxLDL-R_OGTT_ and TBARS-R_OGTT_. The homeostasis model assessment of insulin resistance was used to assess the degree of insulin resistance due to the following formula: HOMA-IR = G-0’ [mmol/L] × Ins-0’ [mU/L]/22.5 [mU × mmol × L^−2^].

### 2.3. Statistical Methods

The statistical analysis was performed using the Statistica 13.3 software (StatSoft Inc., Tulsa, OK, USA) and PQStat Software v.1.8.4 (Poznań, Poland). The Shapiro-Wilk test was used to verify the normality of data distribution. In the absence of a normal distribution, non-parametric tests were used in further analysis. All results were expressed as median and interquartile range. The Mann-Whitney U test was used to assess the significance of differences between the two groups, while the comparisons of many groups were performed using the Kruskal-Wallis test with post hoc analysis of multiple comparisons. Correlations between the studied variables were assessed using Spearman’s R coefficient, and multiple regression analysis was performed if appropriate. Comparative analysis of the tested parameters at 0 and 120 min during OGTT was performed using the Wilcoxon test. The results were considered statistically significant when *p* < 0.05.

The power analysis of comparative tests for many groups in terms of determined oxidative stress parameters ranged from 0.99 to 1.00. The smallest required sample size in each of the four HOMA-IR quartile subgroups considering the parameters oxLDL-0’, oxLD-120’, TBARS-0’, and TBARS-120’ was 7, 13, 9, and 11 subjects, respectively. We recruited 120 people, which resulted in 30 participants in each quartile subgroup.

## 3. Results

The statistical analysis of clinical and biochemical parameters was performed in the entire study population (*n* = 120) and the groups of women (*n* = 46) and men (*n* = 74) as well, and the results are presented in Table 1. The comparison of these groups by the Mann-Whitney U test was also included. Table 2 shows the oxidative stress markers’ characteristics and the comparison between groups.

Differences resulting from the physiological gender otherness were found between the groups of women and men for the waist circumference and HDL-C, while differences in TG and insulinemia at 120 min of OGTT could correspond to the above parameters in the study population. In terms of the assessed parameters of oxidative stress, the groups differed in the concentration of TBARS at 120 min of OGTT (TBARS-120’). For this reason, it was decided to conduct further statistical analysis of the entire study population, regardless of gender.

The correlation analysis considering oxidative stress markers and all clinical and biochemical parameters in the entire study population was performed. Many positive and negative correlations of varying intensity, from weak to very high, were observed. Especially noteworthy are the numerous correlations of all parameters of oxidative stress with insulinemia at 0 (Ins-0’) and 120 min of OGTT (Ins-120’) and the HOMA-IR. This was an inspiration to conduct the analysis in groups distinguished on the basis of HOMA-IR quartiles. Subgroups from H1 to H4 have been separated: H1 (*n* = 30)—HOMA-IR range 1.02–3.00; H2 (*n* = 30)—HOMA-IR range 3.01–4.28; H3 (*n* = 30)—HOMA-IR range 4.29–6.24; H4 (*n* = 30)—HOMA-IR range 6.25–21.68. The characteristics of the subgroups from the H1 to the H4 in terms of the clinical and biochemical parameters and oxidative stress markers with the comparisons between them are presented in Table 3 and Table 4.

**Figure 2:**
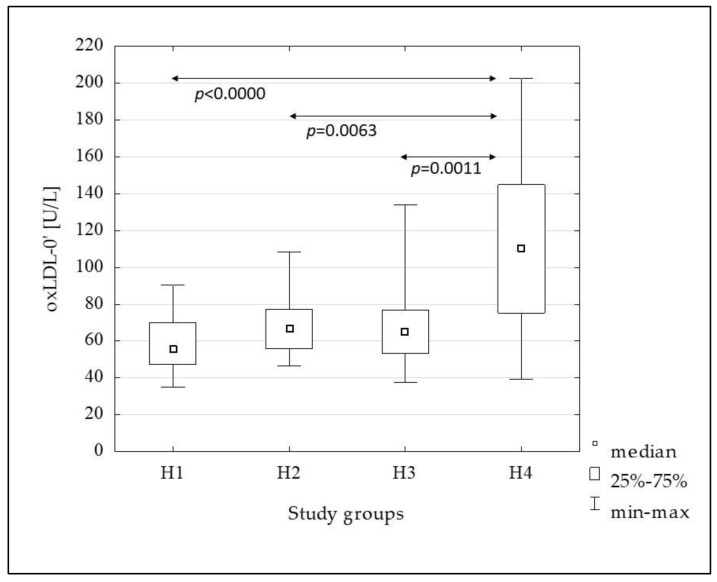
The comparison of the oxLDL-0’ concentration between the H1–H4 subgroups.

**Figure 3:**
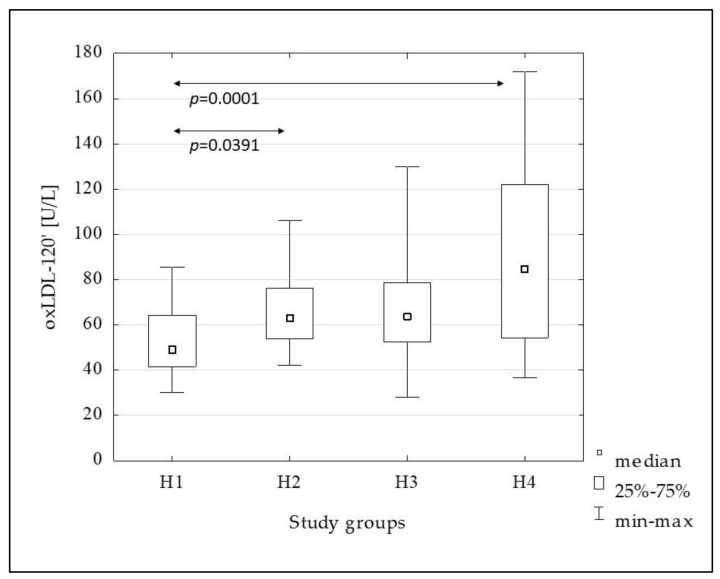
The comparison of the oxLDL-120’ concentration between the H1–H4 subgroups.

**Figure 4:**
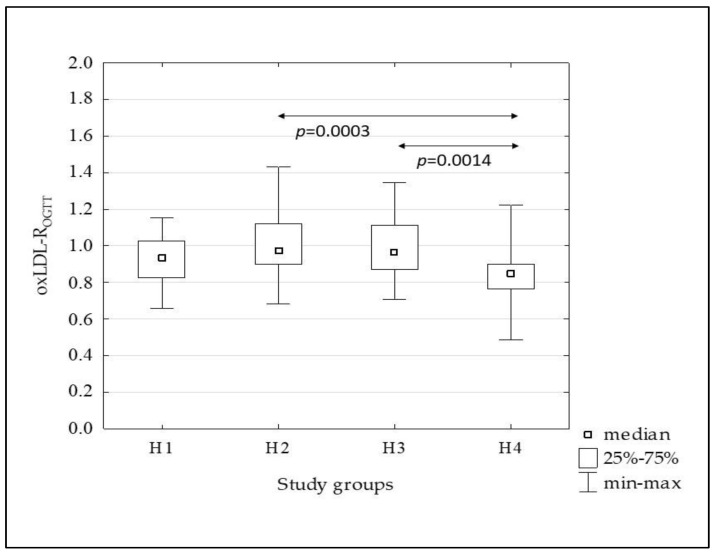
The comparison of the oxLDL-R_OGTT_ ratio between the H1–H4 subgroups.

**Figure 5:**
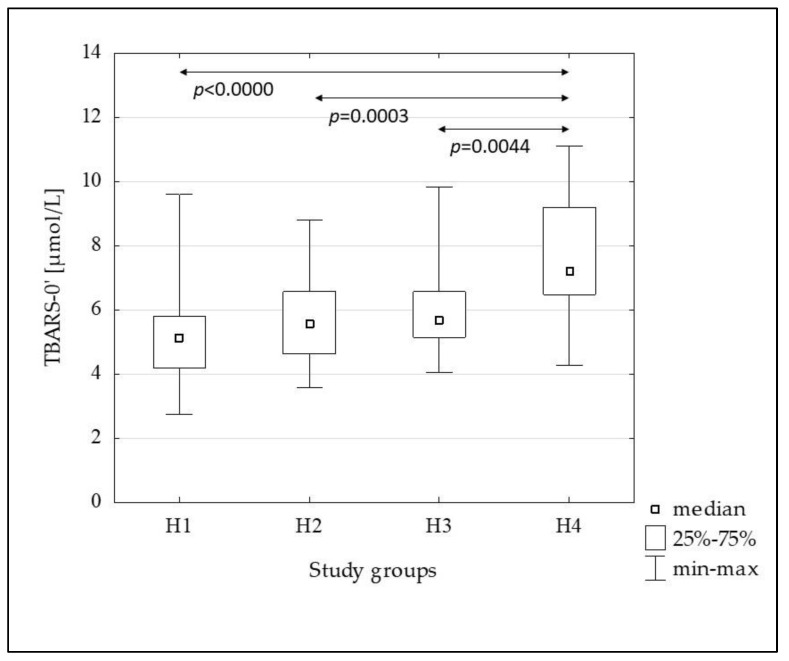
The comparison of the TBARS-0 concentration between the H1–H4 subgroups.

**Figure 6:**
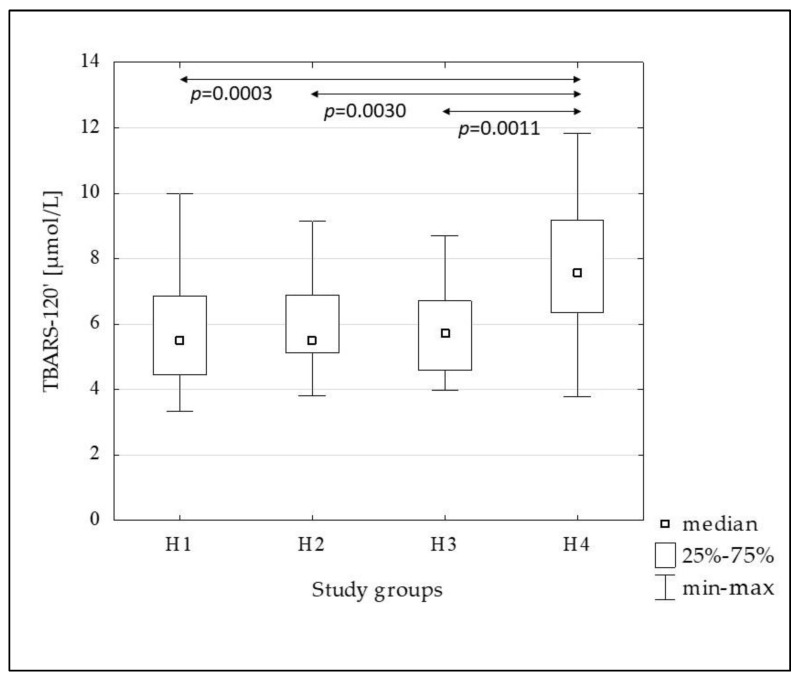
The comparison of the TBARS-120 concentration between the H1–H4 subgroups.

Figure 2, Figure 3, Figure 4, Figure 5 and Figure 6 show the results of comparisons of the analyzed subgroups in terms of oxidative stress markers performed, using the Kruskal-Wallis test with post hoc analysis of multiple comparisons. From the H1 to the H4 groups increasing concentrations of oxLDL were observed both in the fasting state (oxLDL-0’) and at 120 min of OGTT (oxLDL-120’), reaching significantly higher values in the H4 group compared to the others, while the oxLDL-R_OGTT_ ratio decreased from the H2 to the H4 group. TBARS levels also increased, but this was not accompanied by changes in the TBARS-R_OGTT_ ratio.

During the analysis of the correlation between the investigated markers of oxidative stress and all clinical and laboratory parameters in the H1–H4 subgroups, as in the entire population, numerous positive and negative correlations of varying intensity were observed.

The results of the correlation analysis between the oxidative stress markers and parameters of the lipid profile and glycemia in the H1–H4 groups are presented in Table 5. Frequent correlations between oxLDL-0’ and oxLDL-120’ and the parameters of the lipid profile were found. In the H2 group, the concentrations of TBARS-0’ and TBARS-120’ correlated with glycemia at 120 min of OGTT (G-120’), and the strength of the correlation was higher than in the entire population. The results of multiple regression analysis showed that none of the correlations presented (Table 5) was independent of others.

The concentrations of the oxidative stress parameters determined at 0 and 120 min during OGTT showed high and very high mutual correlations both in the entire population and in the subgroups. In addition, correlations between oxLDL-0’ and oxLDL-120’ and TBARS-0’ and/or TBARS-120’ were observed in most of the subgroups.

In the entire study population and subgroups, the analysis of the influence of 75 g of oral glucose load during the OGTT on glycemia, insulinemia, and oxidative stress markers was performed, and the results are presented in Table 6. Changes in the concentrations of all parameters were observed in the entire study population and partly in the individual groups from the H1 to the H4.

## 4. Discussion

Oxidative modification of lipoproteins is one of the earliest elements underlying the extremely complex pathomechanism of cardiovascular diseases. The harmful effect of free radicals on both the lipid and protein parts of lipoproteins leads to a change in their structure and function. Altered lipoproteins do not fulfill their role in lipid metabolism and are taken up by scavenger receptor-presenting cells, which can result in the formation and growth of atherosclerotic plaque [15,16]. The influence of factors modifying the body’s oxidative balance, such as obesity, hyperglycemia, and insulin resistance, may be of significant importance in the development and progression of metabolic disorders and is still a current topic of scientific research. Oxidative stress is also the subject of investigation in various fields of medicine. In addition to metabolic diseases, the importance of the body’s oxidative-antioxidant balance is analyzed in, e.g., pulmonology, neurology, and oncology [17].

When analyzing the relationships between the markers of oxidative stress and the clinical and laboratory data, repeated correlations with Ins-0’ and Ins-120’ and the HOMA-IR were observed in the study population. This was an inspiration to conduct the analysis in the groups distinguished on the basis of the HOMA-IR quartiles. The above data suggest that the HOMA-IR may be useful in categorizing people with increased body mass in the context of cardiovascular risk. Due to the lack of precisely defined cut-off values allowing for the categorization of insulin resistance, the extended clinical usefulness of this index is still a challenge for laboratory medicine.

The mutual correlations between oxLDL-0’ and TBARS-0’ observed in the entire study population, and stronger in people with the highest HOMA-IR values (the H4 group), confirm connections between increased body mass and indicators of insulin resistance in the assessment of oxidative stress markers. Similarly, the correlations between markers of lipid peroxidation and the parameters of the lipid profile and G-120 were more pronounced in the study subgroups. The proposed research model, based on 75 g glucose-provoked metabolic effects with variable post-load glycemia, may correspond to metabolic processes ongoing in our patients day-to-day. In patients with impaired postprandial glucose regulation, mechanisms expressed as insulin resistance and repeated increases in postprandial glycemia may favor a significant overproduction of ROS and increased oxidative stress. Moreover, the presence of a high postprandial glucose concentration also leads to the modification of lipoproteins, including oxLDL, via ROS-mediated oxidation and glycoxidation.

Other authors also drew attention to relationships involving markers of lipid peroxidation. Couillard et al. showed a correlation between the concentration of oxLDL and BMI and the amount of visceral fat in men [18]. Fayez et al. observed higher concentrations of lipid peroxidation products in the blood of obese men, positively correlated with HOMA-IR [19]. This also proves that lipid peroxidation processes in vivo may be enhanced in patients with metabolic disturbances, i.e., insulin resistance, diabetes, and obesity. Regnstrom et al. and Babiy et al. (many years ago) showed that LDLs isolated from the plasma of people with a history of myocardial infarction and diabetes were more susceptible to in vitro oxidation than LDLs isolated from the plasma of healthy people [20,21].

Our results show that the 75 g stimulus causes changes in the concentrations of both assessed oxidative stress parameters in the entire study population. TBARS is a non-specific parameter of the lipid peroxidation process, not only those contained in LDL lipoproteins but also in other fractions of lipoproteins, as well as other plasma and membrane (cellular) lipids. Stimulus-responsive plasma TBARS were increased, which suggests the intensification of lipid oxidation processes under the influence of glucose. Montes-Nieto et al., in 38 people (17 women and 19 men, of whom eight women and nine men were obese), assessed the concentration of TBARS while fasting and after oral administration of glucose (after 1 and 2 h) and fats (after 2 and 4 h) in amounts corresponding to 300 kcal, and in the case of glucose, it was 75 g. The authors observed higher fasting TBARS in the blood of obese men and an increase in TBARS after consuming glucose but not fat and protein. As in the study of Yildirim et al., the concentration of TBARS 60 min after ingestion of 75 g of glucose was higher than after 120 min [22,23]. Thus, it can be assumed that insulin secretion in the regulatory mechanism after glucose ingestion is a factor intensifying oxidative stress. In our study, in the subsequent groups from the H1 to the H4, in which the concentration of insulin was increasing by definition, elevating TBARS-0’ and TBARS-120’ were also observed. In the entire study population, high positive correlations were found between TBARS-0’ and Ins-0’ and HOMA-IR, and average positive correlations between TBARS-120’ and Ins-0’ and HOMA-IR, which were confirmed in the H1 and the H4 groups. Moreover, in the H4 group, Ins-120’ correlated with TBARS-120’, independently of other factors.

In the subsequent groups, from the H1 to the H4, increasing concentrations of oxLDL were observed both in the fasting and post-glucose load state. Additionally, the oxLDL-R_OGTT_ ratio decreased from the H2 to the H4 groups, indicating a decrease in the oxLDL-120’ compared to the fasting value. This suggests an increase in the uptake of these lipoproteins by scavenger receptor-positive cells and/or increased migration into the vascular wall. Certainly, the degree of insulin resistance presented by the H4 group, in which the oxLDL concentration was the highest and the oxLDL-R_OGTT_ ratio was the lowest, is not without significance in comparison to the other groups. Similar to oxLDL, TBARS-0’ and TBARS-120’ increased in subsequent groups, with a significant increase in the H4 group, but in this case, no changes in the TBARS-R_OGTT_ ratio were observed.

We found no study on plasma lipid peroxidation products in overweight and obese subjects in a research model similar to ours. Some scientists, using other biochemical exponents of oxidative stress, different from TBARS and oxLDL, confirmed the increased share of free radicals and derivatives in obesity-related pathology in humans [24,25,26,27,28,29]. The results of studies cited in the discussion also prove a great interest in oxLDL as a marker of lipid peroxidation, especially in the context of cardiovascular risk. Due to the presence and interaction of many modifying factors affecting oxidative balance, and lipid and carbohydrate metabolism, it is difficult to clearly and comprehensively determine the exact contribution of each of them to the development of metabolic diseases.

Our results concern a fragment of overweight and obesity-related pathology, but the awareness that other researchers undertook a thorough study of its individual elements allows us to hope that it will be possible to create a detailed final scenario of metabolic transformations taking into account various factors, including oxidized LDL.

## 5. Limitations and Strength

The study may be limited by the lack of a control group of people with physiological body weight. The idea of this study was to analyze selected parameters during the OGTT only in patients with increased body mass. We are aware that the analysis of the effect of 75 g glucose intake in other clinical states will be a valuable supplement to our research in the future.

Some limitations may also be the greater number of men than women. The reason was fewer eligible overweight women who had not been previously treated at the time of enrollment in the study.

The strength of this study is a unique model for the analysis of selected plasma markers of lipid and lipoprotein peroxidation in the course of insulin resistance, taking into account the OGTT and HOMA-IR quartiles. To some extent, the model may be equivalent to a prospective study on increasing insulin resistance.

## 6. Conclusions

Oral administration of 75 g of glucose may affect markers of oxidative stress measured as oxLDL and TBARS. In patients with increased body mass, increasing insulin resistance may predispose to the intensification of the oxidative modification of plasma lipids and lipoproteins. Personal reduction in the concentration of oxLDL during OGTT, in reference to fasting value (decreased oxLDL-R_OGTT_), suggests increased uptake of modified lipoproteins by scavenger receptor-presenting cells or increased migration to the vascular wall. Various factors may enhance the process of plasma lipid peroxidation: chronic increase in LDL, non-HDL, and total cholesterol, and reduction of HDL cholesterol in the blood, as well as a transient increase of glycemia in the case of insulin resistance.

## Figures and Tables

**Table 1:** The characteristics of the entire study population and the comparison of the groups of women and men. Data are presented as median and interquartile range.

	The Study Population *n* = 120	Women *n* = 46	Men *n* = 74	*p*
Age	53.00 (45.50–61.50)	53.00 (48.00–60.00)	54.00 (44.00–63.00)	ns
BMI [kg/m^2^]	33.00 (29.60–36.10)	34.10 (30.86–36.80)	32.12 (28.71–35.35)	ns
WC [cm]	105.00 (97.00–113.00)	100.00 (95.00–111.00)	106.50 (100.00–117.00)	0.0229
SBP [mmHg]	131.50 (120.00–145.00)	130.00 (120.00–150.00)	133.00 (125.00–145.00)	ns
DBP [mmHg]	86.50 (80.00–90.50)	84.50 (80.00–90.00)	90.00 (80.00–95.00)	ns
T-C [mmol/L]	5.33 (4.73–6.19)	5.35 (4.73–6.21)	5.32 (4.73–6.16)	ns
HDL-C [mmol/L]	1.32 (1.11–1.57)	1.45 (1.24–1.82)	1.26 (1.05–1.49)	0.0020
LDL-C [mmol/L]	3.26 (2.75–4.04)	3.25 (2.74–4.19)	3.30 (2.77–4.03)	ns
nHDL-C [mmol/L]	3.85 (3.41–4.68)	3.84 (3.16–4.97)	3.86 (3.50–4.57)	ns
TG [mmol/L]	1.51 (1.08–2.12)	1.28 (0.90–1.98)	1.61 (1.23–2.34)	0.0262
G-0’ [mmol/L]	5.25 (4.91–5.64)	5.30 (4.92–5.67)	5.22 (4.88–5.64)	ns
G-120’ [mmol/L]	6.38 (4.94–7.93)	6.82 (5.33–7.98)	6.17 (4.89–7.78)	ns
Ins-0’ [mU/L]	18.13 (13.39–25.42)	20.72 (14.64–33.13)	16.75 (13.18–23.79)	ns
Ins-120’ [mU/L]	72.50 (34.65–122.17)	100.24 (45.63–151.72)	60.39 (30.28–100.90)	0.0088
HOMA-IR	4.28 (3.00–6.24)	4.96 (3.02–7.67)	3.83 (2.98–5.82)	ns

*n*—group multiplicity; ns—nonsignificant; BMI—body mass index; WC—waist circumference; SBP—systolic blood pressure; DBP—diastolic blood pressure; T-C—total cholesterol; HDL-C—high-density lipoprotein cholesterol; LDL-C—low-density lipoprotein cholesterol; nHDL-C—non-HDL cholesterol; TG—triglycerides; G-0’—fasting glycemia during OGTT; G-120’—post-load glycemia during OGTT; Ins-0’—fasting insulinemia during OGTT; Ins-120’—post-load insulinemia during OGTT; HOMA-IR—homeostasis model assessment of insulin resistance; OGTT—oral glucose tolerance test.

**Table 2:** The oxidative stress markers in the study population and the comparison of the groups of women and men. Data are presented as median and interquartile range.

	The Study Population *n* = 120	Women *n* = 46	Men *n* = 74	*p*
oxLDL-0’ [U/L]	67.00 (53.15–89.12)	78.91 (54.04–98.00)	64.26 (50.69–77.66)	ns
oxLDL-120’ [U/L]	62.11 (48.80–85.16)	65.60 (49.20–86.40)	60.90 (48.66–78.70)	ns
oxLDL-R_OGTT_	0.92 (0.84–1.03)	0.88 (0.82–1.03)	0.94 (0.85–1.05)	ns
TBARS-0’ [µmol/L]	5.70 (4.85–7.14)	6.00 (5.27–7.32)	5.57 (4.62–6.70)	ns
TBARS-120’ [µmol/L]	6.04 (5.00–7.28)	6.57 (5.30–7.58)	5.56 (4.91–6.96)	0.0207
TBARS-R_OGTT_	1.04 (0.94–1.15)	1.05 (0.98–1.16)	1.03 (0.93–1.14)	ns

*n*—group multiplicity; ns—nonsignificant; oxLDL-0’—fasting oxidized low-density lipoproteins during OGTT; oxLDL-120’—post-load oxidized low-density lipoproteins during OGTT; oxLDL-R_OGTT_—ratio oxLDL-120’/oxLDL-0’; TBARS-0’—fasting thiobarbituric acid reactive substances during OGTT; TBARS-120’—post-load thiobarbituric acid reactive substances during OGTT; TBARS-R_OGTT_—ratio TBARS-120’/TBARS-0’, OGTT—oral glucose tolerance test.

**Table 3:** The comparison of clinical and biochemical parameters in the H1-H4 subgroups. Data are presented as median and interquartile range.

	H1 *n* = 30	H2 *n* = 30	H3 *n* = 30	H4 *n* = 30	*p*
Age	56.50 (50.00–64.00)	53.50 (44.00–60.00)	51.00 (44.00–61.00)	53.00 (40.00–59.00)	ns
BMI [kg/m^2^]	29.23 (26.32–33.70)	30.97 (27.51–32.99)	33.31 (32.70–37.04)	36.95 (33.90–40.80)	<0.0001
WC [cm]	96.00 (90.00–104.00)	104.50 (97.00–110.00)	107.00 (103.00–113.00)	112.00 (103.00–124.00)	<0.0001
SBP [mmHg]	135.50 (120.00–148.00)	130.00 (117.00–142.00)	130.00 (128.00–141.00)	140.00 (125.00–150.00)	ns
DBP [mmHg]	84.00 (71.00–90.00)	83.00 (80.00–90.00)	86.50 (80.00–95.00)	90.00 (80.00–95.00)	ns
T-C [mmoL/L]	5.42 (4.90–6.21)	5.34 (4.87–6.32)	5.37 (4.80–6.34)	4.98 (4.52–6.03)	ns
HDL-C [mmol/L]	1.54 (1.33–1.81)	1.36 (1.17–1.60)	1.21 (1.03–1.48)	1.23 (1.04–1.38)	0.0004
LDL-C [mmol/L]	3.31 (2.93–4.04)	3.49 (3.13–4.12)	3.55 (2.94–4.06)	2.94 (2.56–3.98)	ns
nHDL-C [mmol/L]	3.73 (3.19–4.55)	3.96 (3.62–4.69)	4.31 (3.45–5.12)	3.77 (3.21–4.57)	ns
TG [mmol/L]	1.12 (0.87–1.53)	1.46 (1.08–2.10)	1.74 (1.35–2.40)	1.72 (1.28–2.36)	0.0007
G-0’ [mmol/L]	5.18 (4.65–5.49)	5.12 (4.85–5.46)	5.43 (5.12–5.78)	5.37 (5.02–5.84)	0.0180
G-120’ [mmol/L]	5.29 (4.58–6.80)	6.00 (5.33–7.01)	7.06 (5.28–8.12)	7.81 (5.62–8.95)	0.0042
Ins-0’ [mU/L]	10.10 (8.36–11.36)	15.38 (14.74–16.90)	21.11 (19.71–23.79)	39.09 (32.97–66.55)	<0.0001
Ins-120’ [mU/L]	33.13 (23.69–46.75)	60.02 (38.10–80.84)	88.83 (29.38–123.36)	137.60 (109.34–335.38)	<0.0001
HOMA-IR	2.27 (1.78–2.61)	3.68 (3.27–3.84)	5.10 (4.75–5.62)	9.44 (7.67–14.83)	by definition

*n*—group multiplicity; ns—nonsignificant; BMI—body mass index; WC—waist circumference; SBP—systolic blood pressure; DBP—diastolic blood pressure; T-C—total cholesterol; HDL-C—high-density lipoprotein cholesterol; LDL-C—low-density lipoprotein cholesterol; nHDL-C—non-HDL cholesterol; TG—triglycerides; G-0’—fasting glycemia during OGTT; G-120’—post-load glycemia during OGTT; Ins-0’—fasting insulinemia during OGTT; Ins-120’—post-load insulinemia during OGTT; HOMA-IR—homeostasis model assessment of insulin resistance; OGTT—oral glucose tolerance test.

**Table 4:** The comparison of the oxidative stress markers in the H1-H4 subgroups. Data are presented as median and interquartile range.

	H1 *n* = 30	H2 *n* = 30	H3 *n* = 30	H4 *n* = 30	*p*
oxLDL-0’ [U/L]	55.87 (47.40–70.00)	66.40 (55.80–77.21)	65.15 (53.20–76.80)	110.45 (75.31–145.00)	<0.0001
oxLDL-120’ [U/L]	48.80 (41.55–64.31)	63.08 (53.99–76.26)	63.59 (52.43–78.70)	84.72 (54.40–122.00)	0.0003
oxLDL-R_OGTT_	0.93 (0.82–1.03)	0.98 (0.90–1.12)	0.96 (0.87–1.11)	0.85 (0.76–0.90)	0.0002
TBARS-0’ [µmol/L]	5.12 (4.20–5.79)	5.57 (4.62–6.57)	5.65 (5.14–6.57)	7.21 (6.47–9.19)	<0.0001
TBARS-120’ [µmol/L]	5.49 (4.45–6.85)	5.53 (5.12–6.90)	5.72 (4.59–6.70)	7.57 (6.36–9.16)	0.0001
TBARS-R_OGTT_	1.06 (0.92–1.25)	1.04 (0.98–1.18)	1.02 (0.93–1.13)	1.01 (0.94–1.06)	ns

*n*—group multiplicity; ns—nonsignificant; oxLDL-0’—fasting oxidized low-density lipoproteins during OGTT; oxLDL-120’—post-load oxidized low-density lipoproteins during OGTT; oxLDL-R_OGTT_—ratio oxLDL-120’/oxLDL-0’; TBARS-0’—fasting thiobarbituric acid reactive substances during OGTT; TBARS-120’—post-load thiobarbituric acid reactive substances during OGTT; TBARS-R_OGTT_—ratio TBARS-120’/TBARS-0’, OGTT—oral glucose tolerance test.

**Table 5:** Correlations between the oxidative stress markers and parameters of the lipid profile and glycemia in the H1-H4 groups. The results of significant correlations at *p* < 0.05 are presented. Spearman’s R and *p*-value (R; *p*) are given in parentheses.

	The Study Population *n* = 120	H1 *n* = 30	H2 *n* = 30	H3 *n* = 30	H4 *n* = 30
oxLDL-0’ [U/L]	HDL-C (−0.31; 0.0007) nHDL-C (0.27; 0.0027) TG (0.21; 0.0217)	T-C (0.42; 0.0199) nHDL-C (0.47; 0.0085)			nHDL-C (0.40; 0.0275)
oxLDL-120’ [U/L]	T-C (0.28; 0.0023) HDL-C (−0.29; 0.0014) LDL-C (0.27; 0.0033) nHDL-C (0.40; <0.0000) TG (0.20; 0.0260)	T-C (0.41; 0.0259) LDL-C (0.40; 0.0277) nHDL-C (0.46; 0.0099)	G-120’ (−0.37; 0.0420)	T-C (0.43; 0.0182) nHDL-C (0.40; 0.0295)	LDL-C (0.44; 0.0161) nHDL-C (0.46; 0.0106)
oxLDL-R_OGTT_	T-C (0.21; 0.0224) LDL-C (0.24; 0.0072) nHDL-C (0.20; 0.0307)				
TBARS-0’ [µmol/L]	HDL-C (−0.20; 0.0311) TG (0.27; 0.0024) G-120’ (0.23; 0.0134)		G-120’ (0.52; 0.0035)		
TBARS-120’ [µmol/L]	TG (0.19; 0.0343) G-120’ (0.18; 0.0457)		G-120’ (0.44; 0.0158)		
TBARS-R_OGTT_	T-C (0.19; 0.0331)		T-C (0.41; 0.0238) LDL-C (0.39; 0.0318) nHDL-C (0.46; 0.0111)		

*n*—group multiplicity; T-C—total cholesterol; HDL-C—high-density lipoprotein cholesterol; LDL-C—low-density lipoprotein cholesterol; nHDL-C—non-HDL cholesterol; TG—triglycerides; G 120’—post-load glycemia during OGTT; oxLDL 0’—fasting oxidized low-density lipoproteins during OGTT; oxLDL 120’—post-load oxidized low-density lipoproteins during OGTT; oxLDL-R_OGTT_—ratio oxLDL 120’/oxLDL 0’; TBARS 0’—fasting thiobarbituric acid reactive substances during OGTT; TBARS 120′—post-load thiobarbituric acid reactive substances during OGTT; TBARS-R_OGTT_—ratio TBARS 120’/TBARS 0’, OGTT—oral glucose tolerance test.

**Table 6:** The comparison of the tested parameters during the OGTT (0’ and 120’) in the H1-H4 groups.

Variables	The Study Population *n* = 120	H1 *n* = 30	H2 *n* = 30	H3 *n* = 30	H4 *n* = 30
G-0’ & G-120’	<0.0001	0.0125	0.0036	0.0039	<0.0001
Ins-0’ & Ins-120’	<0.0001	<0.0001	<0.0001	<0.0001	<0.0001
oxLDL-0’ & oxLDL-120’	<0.0001	0.0166	ns	ns	<0.0001
TBARS-0’ & TBARS-120’	0.0127	ns	0.0387	ns	ns

*n*—group multiplicity; ns—nonsignificant; G-0’—fasting glycemia during OGTT; G-120’—post-load glycemia during OGTT; Ins-0’—fasting insulinemia during OGTT; Ins-120’—post-load insulinemia during OGTT; oxLDL-0’—fasting oxidized low-density lipoproteins during OGTT; oxLDL-120’—post-load oxidized low-density lipoproteins during OGTT; TBARS-0’—fasting thiobarbituric acid reactive substances during OGTT; TBARS-120’—post-load thiobarbituric acid reactive substances during OGTT; OGTT—oral glucose tolerance test.

## Data Availability

The original data are available after contact with the corresponding author. The data are not publicly available due to privacy and ethical restrictions.
